# The −174G/C and −572G/C Interleukin 6 Promoter Gene Polymorphisms in Mexican Patients with Rheumatoid Arthritis: A Case-Control Study

**DOI:** 10.1155/2013/959084

**Published:** 2013-09-23

**Authors:** S. A. Zavaleta-Muñiz, B. T. Martín-Márquez, L. Gonzalez-Lopez, N. G. Gonzalez-Montoya, M. L. Díaz-Toscano, J. M. Ponce-Guarneros, A. J. Ruiz-Padilla, M. Vázquez-Del Mercado, M. Maldonado-González, M. Fafutis-Morris, S. E. Flores-Martínez, E. A. Martínez-García, J. I. Gamez-Nava

**Affiliations:** ^1^Unidad de Investigación en Epidemiología Clínica, Centro Médico Nacional de Occidente, Instituto Mexicano del Seguro Social (IMSS), Hospital de Especialidades, 44340 Guadalajara, Mexico; ^2^Doctorado en Ciencias Biomédicas, Orientación en Inmunología, Centro Universitario de Ciencias de la Salud (CUCS), Universidad de Guadalajara, 44340 Guadalajara, Mexico; ^3^Instituto de Investigación en Reumatología y del Sistema Músculo Esquelético, CUCS, Universidad de Guadalajara, 44340 Guadalajara, Mexico; ^4^Departamento de Medicina Interna/Reumatología, Hospital General Regional 110 del Instituto Mexicano del Seguro Social (IMSS), 44710 Guadalajara, Mexico; ^5^Doctorado en Farmacología, Centro Universitario de Ciencias de la Salud (CUCS), Universidad de Guadalajara, 44340 Guadalajara, Mexico; ^6^Coordinación de Posgrado, Centro Universitario de Ciencias de la Salud (CUCS), Universidad de Guadalajara, 44340 Guadalajara, Mexico; ^7^Departamento de Fisiología, CUCS, Universidad de Guadalajara, 44340 Guadalajara, Mexico; ^8^División de Medicina Molecular, Centro de Investigación Biomédica de Occidente (CIBO), IMSS, 44340 Guadalajara, Mexico; ^9^Escuela de Ciencias de la Salud, Universidad del Valle de México, Campus Zapopan, 45010 Zapopan, Mexico

## Abstract

*Objective*. There is a lack of information about the genotype frequencies of IL-6 −174G/C and −572G/C polymorphisms in Mexicans with rheumatoid arthritis (RA). Therefore, the aim of this study was to evaluate the association of the IL-6 −174G/C and −572G/C polymorphisms in Mexican mestizo with RA. *Methods*. We included 137 patients with RA and 102 healthy controls. Patients were assessed for clinical characteristics. IL-6 −174G/C and −572G/C polymorphisms were genotyped using PCR-RFLP analysis. Allele and genotype frequencies and the Hardy-Weinberg equilibrium were computed. Odds ratios (ORs) were computed to identify the risk for RA associated with the presence of GG genotype in comparison with the GC or CC genotypes. *Results*. The genotype −174GG occurred at a higher frequency in cases and controls (77.4% versus 78.4%, *P* = 0.845). We found similar results for the genotype −572GG (54% in patients versus 60.8% in controls, *P* = 0.295). *Conclusions*. This is the first study to evaluate the association of −174G/C and −572G/C polymorphisms of the IL-6 gene with RA in Mexican mestizo patients. These two polymorphisms were not associated with RA in the studied sample. Additional studies are required to evaluate if these IL-6 polymorphisms have relevance to the development of more severe disease.

## 1. Introduction

Rheumatoid arthritis (RA) is a multisystemic autoimmune disease that leads to destruction of the joints and affects 1.6% of the Mexican population [1, 2]. Although the etiology of RA is multifactorial, some genetic factors contribute to individuals' susceptibility to this disease [3, 4]. HLA-DR loci constitute the genetic factor most associated with predisposition to RA [5], although only approximately one-third of the genetic predisposition to RA is explained by HLA. Other gene candidates for susceptibility to RA are cytokine genes. Numerous proinflammatory cytokines, such as tumor necrosis factor alpha (TNF-*α*), interleukin-1 (IL-1), and interleukin-6 (IL-6), are mediators that participate in the inflammatory response and play a role in the pathogenesis of RA [6, 7]. Of these cytokines, IL-6 is considered a key mediator of systemic and localized inflammation in RA, and high levels of IL-6 have been detected in synovial fluid of inflamed joints [8, 9]. Changes in IL-6 level are regulated by multiple factors, including the modulation of variations by polymorphisms within the promoter regions of the IL-6 gene [10, 11]. 

The human IL-6 gene is located on chromosome 7p21. Among the polymorphic sites described in the IL-6 gene promoter, there are two biallelic polymorphisms that may be associated with differences in cytokine production: −572G/C and −174G/C. These polymorphisms consist of a single nucleotide change from guanine (G) to cytosine (C) at positions −572 and −174 in the promoter region, respectively [12, 13].

Some previous reports have investigated a possible association between IL-6 gene polymorphisms and RA or juvenile chronic arthritis (JCA). One study performed in the Caucasian population associated the −174G/C IL-6 gene polymorphism with systemic JCA [14]. However, this association has not been reproduced in adults with RA. In two independent studies performed in Caucasians from Western Europe in Spain [15] and patients from United Kingdom [16], no association was observed between −174G/C polymorphism and RA. Meanwhile, the unique report assessing the relationship between the −572G/C polymorphism and RA was performed in Taiwan by Lo et al. without observing association with this polymorphism [17].

The genotype frequencies of polymorphisms are known to vary according to race or ethnicity. To date, no studies have been performed in Latin America to evaluate whether the −174G/C and −572G/C IL-6 polymorphisms are associated with RA. Therefore, we decided to investigate genotype and allele frequencies of these two polymorphisms in Mexican mestizo patients with RA and to compare these frequencies with those observed in controls of similar ethnic origin.

## 2. Materials and Methods

### 2.1. Design

This is a case-control study.

### 2.2. Clinical Setting

The study was performed from January 2009 to January 2011 in an outpatient rheumatology clinic of a secondary-care hospital in Guadalajara, Mexico (Hospital General Regional 110, del Instituto Mexicano del Seguro Social, IMSS). The RA group was comprised of patients who met the 1987 revised American College of Rheumatology (ACR) criteria. Healthy subjects in a similar age range were selected as controls. Study participants (both patients with RA and healthy controls) were included if they were Mexican mestizo (defined as having at least two generations of ancestors born in Western Mexico); only one person per family (18 years of age or older) was recruited. Individuals who were adopted or of unknown ancestral origin were excluded from both study groups. Healthy control participants were also excluded if they had a family history of rheumatic inflammatory disease; patients with overlapping syndrome in RA were also excluded.

### 2.3. Clinical Assessment

All patients and controls were interviewed to assess sociodemographic characteristics. Patients with RA were evaluated regarding functionality using the Health Assessment Questionnaire Disability Index (HAQ-DI) and regarding disease severity using the 28-item Disease Activity Score (DAS-28) and history of RA medication.

### 2.4. Determination of Polymorphisms

Genomic DNA was isolated from white blood cell using the standard protocol. To identify the −174G/C polymorphism, a 198 bp fragment was amplified using the forward primer 5′ TGACTTCAGCTTTACTCTTTGT 3′and the reverse primer 5′ CTGATTGGAAACCTTATTAAG 3′; the conditions of the reaction were as follows: denaturalization at 94°C for 60 seconds, followed by 35 cycles of annealing at 53°C for 1 minute and 20 seconds, extension at 72°C for 1 minute and 20 seconds, and a final elongation step extension at 72°C for 5 minutes. To identify the −572G/C polymorphism, we amplified a 163 bp using the forward primer 5′ GGAGACGCCTTGAAGTAACTGC 3′ and the reverse primer 5′ GAGTTTCCTCTGACTCCATCGCAG 3′; the conditions of the reaction were as follows: denaturalization at 94°C for 60 seconds, followed by 35 cycles of annealing at 55°C for 60 seconds, extension at 72°C for 60 seconds, and a final extension step at 72°C for 60 seconds. Polymerase chain reactions (PCRs) were performed using a PCR thermal cycler (ESCO). PCR products were digested with SfaNI enzyme and BsrBI enzyme to identify the −174/G/C and −572G/C polymorphisms, respectively, according to the manufacturer's instructions.

Each PCR product was electrophoresed on a 6% polyacrylamide gel stained with silver nitrate. The resultant genotypes for both polymorphisms were classified as the nonexcisable homozygote allele (CC), the excisable homozygote allele (GG), and the heterozygote allele (CG). After enzymatic digestion of the amplified fragment for the −174G/C polymorphism, we were able to identify the different GG (148 pb and 50 bp), GC (198 bp, 148 bp, and 50 bp), and CC (198 bp) genotypes. For the −572G/C polymorphism, we identified the different genotypes as 102 bp and 61 bp fragments for the GG genotype; 163 bp, 102 bp, and 61 bp fragments for the GC genotype; a 163 bp fragment for the CC genotype.

### 2.5. IL-6 Measurement

IL-6 in serum was measured with an enzyme-linked immunosorbent assay using commercial kits (R&D Systems, Minneapolis, MN, USA). The detection range provided is 3.12 to 300 pg/mL, and the minimum detectable dose (MDD) of IL-6 is typically less than 0.70 pg/mL.

### 2.6. Statistical Analysis

The allele and genotype frequencies of both polymorphisms were obtained by direct counting. Hardy-Weinberg equilibrium was evaluated for the control group using the Chi-squared test. Genotype and allele frequencies between RA and controls were compared using the Chi-squared test or Fisher's exact test if required.

Odds ratios (ORs) and their 95% confidence intervals (95% CI) were computed to identify the RA risk associated with the presence of GG genotype in comparison with the GC or CC genotypes (used as a referent). A similar strategy was used to identify the risk associated with the G allele. The *P* value was set at 0.05. All statistical analysis was performed using SPSS version 8.0 or EPI INFO version 6.04.

### 2.7. Ethics

This study was approved by a research committee from the participant center R-2009-1301-78. All study participants voluntarily provided written informed consent. All procedures in the protocol were performed according to the guidelines of the Declaration of Helsinki.

## 3. Results

We included 137 patients with RA and 102 healthy controls. Comparison in selected characteristics between patients with rheumatoid arthritis and healthy controls is shown in Table 1. No significant differences were observed between the study groups with regard to age (*P* = 0.191) or sex (*P* = 0.076). Serum levels of IL-6 were higher in patients with RA than in the control group (mean of levels 10.9 versus 1.14 pg/mL, resp., *P* < 0.001). 

Table 2 describes the clinical and laboratory characteristics in patients with RA. They had a mean disease duration of 10 years, a mean DAS-28 of 4.1, and a mean HAQ-Di of 0.67. Mean titles for rheumatoid factor were 200.5 ± 384.2 UI/mL, for C-reactive protein 17.0 ± 25.9 mg/L, and for ESR 30.7 ± 12.6 mm/hr. At the time of the study, 80.3% of patients were taking methotrexate, 21.9% chloroquine, 18.9% leflunomide, and only 13.8% received biologic agents.

For the control group, both −174G/C and −572G/C IL-6 polymorphisms were in Hardy-Weinberg equilibrium (*P* = 0.52 and *P* = 0.31, resp.).

Table 3 shows the genotype and allele frequencies as well as the comparison between patients and controls of both polymorphisms. There were no statistical differences between the study groups.

## 4. Discussion

The results of the present study identified that, for both −174G/C and −572G/C polymorphisms, the GG genotype is the most frequently observed in the Mexican mestizo population among patients with RA and healthy controls. We observed no differences in allele or genotype frequencies of these polymorphisms between RA and controls.

Our findings regarding GG being the most frequently encountered genotype for the −174G/C polymorphism in patients with RA are similar to those observed in Spain by Pascual et al. [15]. This group did not find an association between this polymorphism and RA; however, it must be noted that they obtained a genotype frequency different from ours (GG genotype 46% versus 77.4%, GC 44.2% versus 21.9%, and CC 9.8% versus 0.7%, resp.). In another contrasting study by Marinou et al. in the United Kingdom [16], the GC genotype was the most frequent, although not associated with RA (GC genotype 51.8% versus 21.9% in ours study). This finding underlines the relevance of racial mixing to the polymorphism frequency in Mexican mestizos.

For the polymorphism −572G/C, only Lo et al. in Taiwan [17] have reported a comparison between genotype frequencies in patients with RA and controls. They found a genotype distribution with wide differences in frequency compared with our study and observed no association between RA and any particular genotype. In our study population, the GG genotype was the most frequently observed, contrasting with the results described by Lo et al., (54% versus 4.5%, resp.). 

Our results show the variability in genotype frequencies for both polymorphisms observed in Mexican patients and controls. It has been observed that polymorphisms in the promoter region of the IL-6 gene may be responsible for changes in the expression of IL-6, which could in turn lead to greater inflammation and thus affect the clinical status of RA patients [12]. Additional studies should include the evaluation of whether changes in serum levels of IL-6 are associated with these genotypes; however, this aim was beyond the scope of the present study.

One strength of our study is the use of a carefully defined population in which patients and controls were Mexican mestizos with a family history of living for at least three generations in the western region of Mexico, and healthy controls were selected to closely match our RA patients regarding age and sex, minimizing these confounding factors present in other studies. Nevertheless, because the Mexican population features great ethnic diversity, therefore one limitation of the present study is that we limited the inclusion to subjects born in Western region of the country. We do not know whether our results are generalizable to other regions. 

In conclusion, this is the first study to evaluate the association of −174G/C and −572G/C polymorphisms of the IL-6 gene with RA in Mexican mestizo patients. These results are relevant to improving the understanding of the genetic factors associated with RA in patients of this ethnicity. Although these two polymorphisms were not associated with RA, additional studies are required to evaluate the relevance of these IL-6 polymorphisms to the development of more severe disease.

## Figures and Tables

**Table 1:** Comparison in selected characteristics between patients with rheumatoid arthritis and healthy controls.

Characteristics	Cases with RA *n* = 137	Controls *n* = 102	*P*
Age (years), mean ± SD	50 ± 9	48 ± 10	0.191
Females (%)	135 (98.5)	96 (94.1)	0.076
Serum IL-6 (pg/mL)	10.9 ± 17.9	1.14 ± 3.4	<0.001

Quantitative variables are expressed as means ± standard deviations (SD) and qualitative variables as frequencies and percentages (%) as noted. Abbreviations: RA: rheumatoid arthritis, IL-6: interleukin 6.

Comparisons between means were performed with unpaired Student's *t*-test. Comparisons between proportions were performed with Chi-squared test.

**Table 2:** Clinical and laboratory characteristics of patients with RA.

Characteristic	RA *n* = 137
Disease duration (years), mean ± SD	10 ± 7.9
Females (%)	135 (98.5)
DAS-28, mean ± SD	4.1 ± 1.5
HAQ-DI, mean ± SD	0.67 ± 0.6
Laboratory findings	
Serum Rheumatoid factor (UI/L), mean ± SD	200.5 ± 384.9
C-reactive protein (mg/L), mean ± SD	17.0 ± 25.9
Erythrocyte sedimentation rate (mm/h), mean ± SD	30.7 ± 12.6

Quantitative variables are expressed as means ± standard deviations (SD) and qualitative variables as frequencies and percentages (%) as noted. Abbreviations: RA: rheumatoid arthritis, DAS-28: disease activity score of 28 joints, HAQ-DI: Health Assessment Questionnaire Disability Index.

**Table 3:** Comparison in genotype and allele frequencies of −174G/C and −572G/C polymorphisms between the groups with rheumatoid arthritis and controls.

Genotype	Polymorphism −174G/C			*P*	Polymorphism −572G/C			*P*
	RA *n* (%) *n* = 137	Controls *n* (%) *n* = 102	OR (95% CI)		RA *n* (%) *n* = 137	Controls *n* (%) *n* = 102	OR (95% CI)	
GG	106 (77.4)	80 (78.4)	0.94	0.845	74 (54)	62 (60.8)	0.76	0.295
GC	30 (21.9)	20 (19.6)	(0.48–1.82)		58 (42.3)	37 (36.3)	(0.44–1.32)	
CC	1 (0.7)	2 (2)			5 (3.6)	3 (2.9)		
Allele								
G	242 (88.3)	180 (88.2)	1.01	0.976	206 (75.2)	161 (78.9)	0.81	0.338
C	32 (11.7)	24(11.8)	(0.57–1.8)		68 (24.8)	43 (21.1)	(0.1–1.28)	

RA: Rheumatoid arthritis; OR: odds ratio; 95% CI: confidence interval. For genotype, OR was computed using GG as a risk factor and GC + CC as the referent.
